# Multi-mode adaptive control strategy for a lower limb rehabilitation robot

**DOI:** 10.3389/fbioe.2024.1392599

**Published:** 2024-05-16

**Authors:** Xu Liang, Yuchen Yan, Shenghua Dai, Zhao Guo, Zheng Li, Shengda Liu, Tingting Su

**Affiliations:** ^1^ School of Automation and Intelligence, Beijing Jiaotong University, Beijing, China; ^2^ Department of Mechanical and Electrical Engineering, North China University of Technology, Beijing, China; ^3^ School of Power and Mechanical Engineering, Wuhan University, Wuhan, China; ^4^ Faculty of Medicine, The Chinese University of Hong Kong, Shatin, China; ^5^ Institute of Automation, Chinese Academy of Sciences, Beijing, China; ^6^ Faculty of Information Technology, Beijing University of Technology, Beijing, China

**Keywords:** impedance control, rehabilitation robot, multi-mode adaptive control, human–robot interaction, rehabilitation training strategy

## Abstract

Different patients have different rehabilitation requirements. It is essential to ensure the safety and comfort of patients at different recovery stages during rehabilitation training. This study proposes a multi-mode adaptive control method to achieve a safe and compliant rehabilitation training strategy. First, patients’ motion intention and motor ability are evaluated based on the average human–robot interaction force per task cycle. Second, three kinds of rehabilitation training modes—robot-dominant, patient-dominant, and safety-stop—are established, and the adaptive controller can dexterously switch between the three training modes. In the robot-dominant mode, based on the motion errors, the patient’s motor ability, and motion intention, the controller can adaptively adjust its assistance level and impedance parameters to help patients complete rehabilitation tasks and encourage them to actively participate. In the patient-dominant mode, the controller only adjusts the training speed. When the trajectory error is too large, the controller switches to the safety-stop mode to ensure patient safety. The stabilities of the adaptive controller under three training modes are then proven using Lyapunov theory. Finally, the effectiveness of the multi-mode adaptive controller is verified by simulation results.

## 1 Introduction

In recent years, the number of patients with movement disorders caused by stroke and spinal cord injury has increased rapidly, as has the corresponding rehabilitation demand. Traditional rehabilitation strategies rely on therapists to help patients participate in training, and there are some problems such as long rehabilitation cycle and low efficiency of rehabilitation which make it difficult to meet the growing recovery needs (Luo et al., 2019). As a new way of rehabilitation training, rehabilitation robots can effectively save medical resources and improve the efficiency of rehabilitation training. Therefore, this has received wide attention and recognition (Adhikari et al., 2023).

The control method plays a crucial role in the rehabilitation effect (Zhou et al., 2021) as the patient has been interacting with the robot during the training process. Traditional control methods may subject the patient to excessive torque, which increases the risk of secondary injury. In contrast, control methods based on human–robot interactive information can have good rehabilitation training effects (Guo et al., 2021). Such methods can not only effectively avoid potential injuries but also help improve recovery. Therefore, it is important to design a safe, natural, and compliant human–robot interaction control method for rehabilitation robot systems (Masengo et al., 2023; Bergmann et al., 2023; Li Z. et al., 2024; Lu et al., 2023).

For patients with weak motor ability, rehabilitation robots should provide enough assistive force to help complete training tasks. However, too much assistance may make patients slack off, and too little assistance will not help patients implement training tasks—both may reduce rehabilitation effects. In order to realize efficient rehabilitation training, human–robot interaction methods need to follow the assisted-as-need (AAN) principle (Li N. et al., 2024). At present, impedance control is usually used to implement the AAN strategy (Han et al., 2023). Mao et al. (2015) established a force field controller which constructs a virtual tunnel with impedance characteristics around the desired trajectory to assist the patient’s movement. Jamwal et al. (2016) built an impedance controller for an ankle robot to assist patient compliance. Due to individual differences, it is difficult to obtain optimal impedance parameters. In addition, the interaction force and motion speed change over time, and fixed impedance parameters usually cannot meet the practical needs. The dynamic relationship between motion and interaction force can be adjusted according to the actual task by using time-varying impedance control; thus, good dynamic interaction performance can be achieved (Liang et al., 2022). Asl et al. (2020) constructed an AAN impedance controller which utilizes velocity tracking errors to adjust impedance parameters online. However, only the damping parameter is adjusted in this study, and its adaptive adjustment ability is relatively limited. Han et al. (2023) proposed an AAN control strategy for rehabilitation robots based on patients’ motor intention and task performance. The learning efficiency of impedance parameters and the auxiliary level were adaptively adjusted according to the assessment results of interaction force and patient performance. The experimental results show that this method can motivate patients to increase their engagement.

For patients with a partial recovery of motor function or strong motor ability, interference with their movement should be reduced to provide sufficient freedom of movement (Han et al., 2023; Zhang and Cheah, 2015). Higher freedom of movement does not mean that patients can move without restriction. When the position and speed of robots reach a certain level, patients may be exposed to the potential risk of secondary injury (Gao et al., 2023). To ensure patient safety, control methods should have safety features such as emergency stops or motion position limitations.

To meet the needs of patients at different recovery stages and ensure their safety, multi-mode control strategies have been proposed (Zhang and Cheah, 2015; Li et al., 2021; Yang et al., 2023; Xu et al., 2019; Li et al., 2017a,b). Zhang and Cheah (2015) proposed a multi-mode control method for upper limb rehabilitation robots. The training mode is chosen based on the position error to realize safety assistance. Li et al. (2021) and Yang et al. (2023) also designed multi-mode control strategies and switched control modes according to the tracking error. These methods switch control modes according to the position errors, which will partly limit the movement freedom of patients with strong motor ability. To solve this problem, a patient’s bioelectrical or interactive force signals can be used as the basis for switching training modes. Xu et al. (2019) proposed a multi-mode adaptive control strategy for a sitting lower limb rehabilitation robot. The human–robot interaction torque is estimated by using an EMG-driven impedance model. Based on the estimated human–robot interaction torque, a smooth transition between the robot-dominant and human-dominant modes can be achieved. Compared with bioelectrical signals, interaction force signals are more reliable. Li et al. (2017a) proposed an adaptive control method to smoothly switch the training modes between robot- and human-dominant modes based on the human–robot interaction force to realize safe interaction between humans and robots. Since this method ignores trajectory errors, the trajectory errors in the human-dominant mode may be large, which will lead to a reduction in the training effect. In the multi-mode control strategy, relying on only a single signal cannot provide the most suitable rehabilitation training mode for patients. Li et al. (2017b) proposed a multi-mode control strategy in which the tracking error and human-robot interaction force are taken as the basis for mode switching. Based on the tracking error, the controller can switch flexibly between human- and robot-dominant modes. When the human–robot interaction force exceeds the safety threshold, the controller will switch to the safety-stop mode to ensure the patient’s safety. This method still uses the tracking error as the basis for switching between human- and robot-dominated modes, which will also limit the movement freedom of patients with strong motor ability. In addition, the interaction force signals cannot fully indicate the patients’ motor ability.

To solve such problems, a multi-mode adaptive control strategy for repetitive rehabilitation tasks is here proposed. The human–robot interaction force evaluation factor is introduced to assess a patient’s motor ability and motor intention online (Han et al., 2023). Based on the evaluation result of the patient’s motor ability and trajectory errors, the training mode can be freely switched between robot-dominant, patient-dominant, and safety-stop modes. In the robot-dominant mode, the robot’s assistance level and the learning efficiency of impedance parameters are periodically adjusted according to the trajectory error, speed error, the assessed motor ability, and the motion intention, so as to provide appropriate assistance for patients with different motor abilities. In the patient-dominant mode, the controller allows the patient to modify the reference speed so that patients with higher motor ability have enough freedom of movement. When the trajectory error exceeds the safe range, it switches to safety-stop mode to ensure patient safety. The proposed method is not only suitable for patients at different stages of recovery and with different motor abilities but can also stimulate their enthusiasm to participate in rehabilitation training, further enhancing the rehabilitation effect.

## 2 Dynamic model of the human–robot hybrid system

During the rehabilitation training, the lower limb rehabilitation robot is in close contact with the patients’ affected limb, forming a human–robot hybrid system. The hybrid system’s dynamic model is shown as Eq. 1.

$$M\left(q\right)\ddot{q}+C\left(q,\dot{q}\right)\dot{q}+G\left(q\right)=\tau_{r}+\tau_{h},$$ (1)

where $q={\left[q_{1}\cdots q_{i}\right]}^{\text{T}}$ represents the robot’s joint angle, and *i* denotes the number of the robot’s joints. $\dot{q}$ and $\ddot{q}$ represent the angular speed and angular acceleration, respectively. $M\left(q\right)$ , $C\left(q,\dot{q}\right)$ and $G\left(q\right)$ denote the inertia matrix, the Coriolis and centrifugal matrix, and the gravity vector, respectively. *τ*_*r*_ and *τ*_*h*_ respectively represent the actuation torque and interaction torque exerted by patient. In this paper, the interaction force *F*_*h*_ is exerted on the robot end, give by Eq. 2.

$$\tau_{h}=J^{\text{T}}\left(q\right)F_{h},$$ (2)

where *J*(*q*) represents the Jacobian matrix.

## 3 Multi-mode control method

The functions and designs of the three control modes are briefly introduced in this section. For repetitive tasks, when the patient does not have enough motor ability to independently complete the training task, the robot-dominant mode runs. Adaptive assistance is then provided according to the patient’s motor ability and motion intention. For patients with weak motor ability, the assistance level will be periodically increased. For patients with a certain motor ability but who cannot yet complete the task independently, the assistance intensity will reduce appropriately to encourage more active participation in the training task. When the patient has recovered part of the motor function and can complete the training task independently, the patient-dominant mode runs. In this case, only movement speed is adjusted to provide the patient with a high degree of freedom of movement. When the patient’s movement is abnormal or the task is too difficult, the robot’s trajectory may exceed the safe range. In this case, the safety-stop mode runs to ensure patient safety.

### 3.1 Design of human–robot interaction force evaluation factor and mode shift factor

According to the functional requirements of the three control modes, a unified control law is established that includes the reference term, impedance learning term, sliding term, and compensation term, as shown below.

$$\tau_{r}=\underset{reference\;term}{\underbrace{M{\ddot{q}}_{\mathrm{ref}}+C{\dot{q}}_{\mathrm{ref}}+G}}+\underset{impedance\;learning\;term}{\underbrace{\alpha \left(K\left(t\right)e+D\left(t\right)\dot{e}\right)}}+\underset{sliding\;term}{\underbrace{L\left(t\right)s}}+\underset{compensation\;term}{\underbrace{\tau_{c}\left(t\right)}}$$ (3)

where *M*, *C*, and *G* are abbreviations of $M\left(q\right),C\left(q,\dot{q}\right)$ , and $G\left(q\right)$ , respectively. *K*(*t*) and *D*(*t*) denote variable stiffness and damping, respectively. *L*(*t*) denotes the sliding control gain, while *e* = *q*_*d*_ − *q* represents the trajectory error between the desired *q*_*d*_ and actual trajectory *q*. $s={\dot{q}}_{\mathrm{ref}}-\dot{q}$ denotes the sliding vector. ${\dot{q}}_{\mathrm{ref}}=\left(1-\beta \right)\left({\dot{q}}_{d}+\alpha Ae+\left(1-\alpha \right){\dot{q}}_{h}\right)$ denotes the reference speed, where *A* is a symmetric positive definite matrix, ${\dot{q}}_{h}$ is the modified speed determined by the interaction torque, *α* is the robot–patient mode shift factor determined by the patients’ motor ability, and *β* is the stop-mode shift factor, determined by the trajectory error. Before analyzing the change pattern of these two mode shift factors, the human–robot interaction force evaluation factors $r_{j}^{\mathrm{par}}$ and $r_{j}^{\mathrm{ort}}$ are introduced to assess the patients’ motor ability and motion intention (Han et al., 2023) as shown in Eqs 4, 5.

$$r_{j}^{\mathrm{par}}=\frac{1}{T}\int_{t_{j-1}}^{t_{j}}F_{h}^{\mathrm{par}}dt=\frac{1}{T}\int_{t_{j-1}}^{t_{j}}F_{h}^{\text{T}}\frac{{\left[{\dot{x}}_{d},{\dot{y}}_{d}\right]}^{\text{T}}}{\sqrt{{\dot{x}}_{d}^{2}+{\dot{y}}_{d}^{2}}}dt,$$ (4)

$$r_{j}^{\mathrm{ort}}=\frac{1}{T}\int_{t_{j-1}}^{t_{j}}F_{h}^{\mathrm{ort}}dt=\frac{1}{T}\int_{t_{j-1}}^{t_{j}}F_{h}^{\text{T}}\frac{{\left[-{\dot{y}}_{d},{\dot{x}}_{d}\right]}^{\text{T}}}{\sqrt{{\dot{x}}_{d}^{2}+{\dot{y}}_{d}^{2}}}dt,$$ (5)

where $F_{h}^{\mathrm{par}}$ represents the human–robot interaction force parallel to the desired trajectory. $F_{h}^{\mathrm{ort}}$ represents the human–robot interaction force perpendicular to the desired trajectory. $\left({\dot{x}}_{d},{\dot{y}}_{d}\right)$ represents the desired speed at the robot end. *F*_*h*_ can be obtained by the interaction force estimation methods (Lu et al., 2023; Liang et al., 2023). *j* denotes the *j*th training task, *T* denotes the task period. *t*_*j*_, and *t*_*j*−1_ represent the initial moments of the *j*th and (*j* − 1)th task, respectively.

When $r_{j}^{\mathrm{par}}$ is positive, the patient’s movement speed is greater than the desired speed, and the robot is driven by the patient along the desired trajectory. On the other hand, when $r_{j}^{\mathrm{par}}$ is negative, the patient is driven by the robot along the desired trajectory. $\left|r_{j}^{\mathrm{ort}}\right|>0$ means that the patient intends to deviate from the desired trajectory. The greater the $r_{j}^{\mathrm{par}}$ , the stronger the patient’s motor ability. The larger $\left|r_{j}^{\mathrm{ort}}\right|$ is, the stronger the patient’s intention to move away from the desired trajectory.

The change pattern of *α* and *β* is designed as follows:

$$\beta =\left\{\begin{array}{cc} 1,\; & \left\|e\right\|\in \left(a,+\infty \right)\\ 0,\; & \left\|e\right\|\in \left[0,a\right] \end{array}\right.,$$ (6)

$$\alpha =\left\{\begin{array}{cc} 1,\; & \beta =0\;and\;r_{j}^{\mathrm{par}}\in \left(-\infty ,c\right]\\ 0,\; & \beta =0\;and\;r_{j}^{\mathrm{par}}\in \left(c,+\infty \right)\\ 0,\; & \beta =1 \end{array}\right.,$$ (7)

where *a* and *c* are given values. $\left\|e\right\|$ denotes the Euclidean norm of *e*. If $\left\|e\right\|>a$ , then the task is too difficult or the patient’s movement is abnormal, which will lead to excessive trajectory errors or even secondary injury to the patient. The controller will then switch to safety-stop mode to ensure patient safety. If $\left\|e\right\|\leq a$ , then the patient is able to complete training task either with the robot’s assistance or independently. The controller will then switch to either robot-dominant or patient-dominant mode based on the value of *α*. If $r_{j}^{\mathrm{par}}>c$ , then the patient can complete the task independently, and it will switch to patient-dominant mode. If $r_{j}^{\mathrm{par}}\leq c$ , the patient’s motor ability is insufficient to complete the training task, and it will switch to the robot-dominant mode. In practice, *c* can be set to a constant close to 0. If the patient has good control over the affected limb, *c* can be slightly reduced, allowing the controller to easily enter and maintain the patient-dominant mode. If the patient has poor control over the affected limb, *c* can be slightly increased so that the controller is always in robot-dominant mode so that the robot can help the patient complete the training task and correct their wrong movements.

**FIGURE 1:**
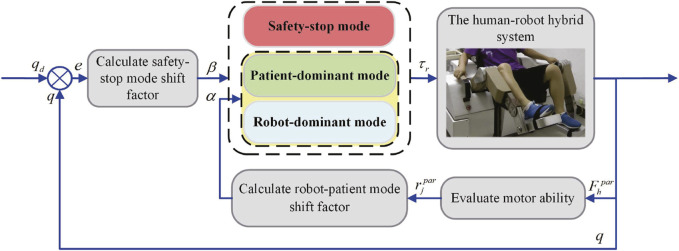
Multi-mode adaptive control block diagram.

**FIGURE 2:**
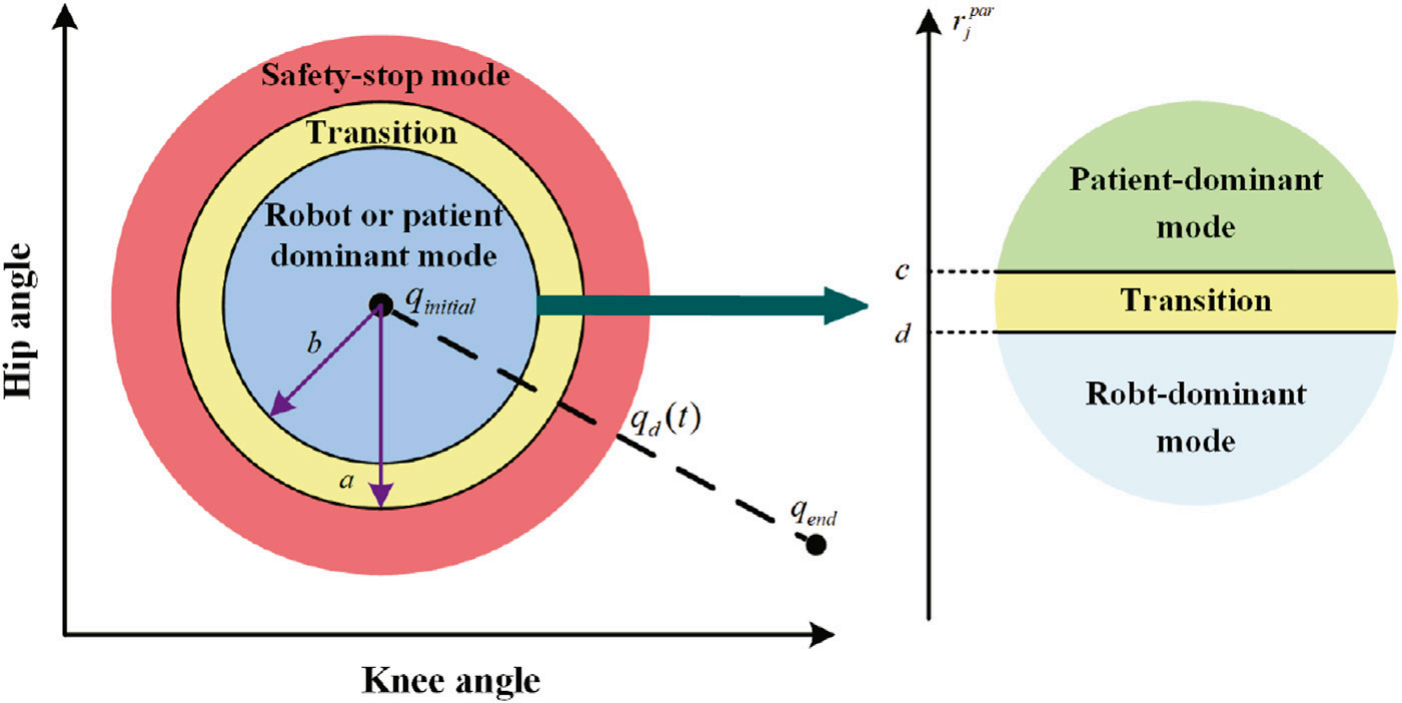
Training modes.

The control diagram is shown in Figure 1. To ensure patient safety, the safety-stop mode has the highest priority among the three modes, which is reasonable in practical applications. To ensure the smoothness of the mode switching process, transition intervals are added to Eqs 6, 7, and then the change pattern of *α* and *β* is modified as Eqs 8, 9.

$$\beta =\left\{\begin{array}{cc} 1,\; & \left\|e\right\|\in \left(a,+\infty \right)\\ \frac{{\left[{\left({\left\|e\right\|}^{2}-a^{2}\right)}^{4}-{\left(b^{2}-a^{2}\right)}^{4}\right]}^{4}}{{\left(b^{2}-a^{2}\right)}^{16}},\; & \left\|e\right\|\in \left(b,a\right]\\ 0,\; & \left\|e\right\|\in \left[0,b\right], \end{array}\right.$$ (8)

$$\alpha =\left\{\begin{array}{cc} 1,\; & \beta \neq 1\;and\;r_{j}^{\mathrm{par}}\in \left(-\infty ,d\right]\\ 1-\sin^{2}\left(\frac{\left(r_{j}^{\mathrm{par}}-d\right)\pi }{2\left(c-d\right)}\right),\; & \beta \neq 1\;and\;r_{j}^{\mathrm{par}}\in \left(d,c\right]\\ 0,\; & \beta \neq 1\;and\;r_{j}^{\mathrm{par}}\in \left(c,+\infty \right)\\ 0,\; & \beta =1, \end{array}\right.$$ (9)

where *b* and *d* are given values. The modified *α* and *β* change smoothly as $r_{j}^{\mathrm{par}}$ and *e* change. According to the patient’s motor ability and trajectory error, the controller switches freely between the three modes (Figure 2). However, $r_{j}^{\mathrm{par}}$ is periodically adjusted, and it will cause *α* to be discontinuous in time. When *α* changes at *t*_1_, the changed *α* is expressed as *α*_1_ = *α*(*t*_1_), and we have

$$\alpha \left(t\right)=\begin{array}{c} \alpha_{s}+\left(\alpha_{1}-\alpha_{s}\right)\sin^{2}\left(\frac{\left(t-t_{1}\right)\pi }{2T_{\mathrm{smo}}}\right)t\in \left[t_{1},t_{1}+T_{\mathrm{smo}}\right] \end{array},$$ (10)

where *α*_*s*_ = *α*(*t*_1_ − *t*_*s*_), *t*_*s*_ is the sampling time, and *T*_*smo*_ is the smoothing time. Thus, *α* is smooth in time.

Although Eq. 10 can ensure the continuity of *α*, *T*_*smo*_ may also cause a lag in mode switching. Therefore, the value of *T*_*smo*_ should not be too large in practical applications.

### 3.2 The Robot-dominant mode

When *α* = 1, *β* = 0, the controller is in the robot-dominant mode. In this mode, the human–robot interaction torque is described as Eq. 11 (Han et al., 2023; Yang et al., 2011):

$$\tau_{h}\left(t\right)=\tau_{0}\left(t\right)+K_{h}\left(t\right)e+D_{h}\left(t\right)\dot{e},$$ (11)

where the stiffness parameters $K_{h}\left(t\right)$ , damping parameters $D_{h}\left(t\right)$ , and compensating torque $\tau_{0}\left(t\right)$ are assumed to vary with time. The minimum quantities of stiffness, damping, and compensating torque are assumed to be *K*_*m*_(*t*), *D*_*m*_(*t*), and $\tau_{m}\left(t\right)$ , respectively, and

$$\begin{array}{c} \int_{t-T}^{t}-\left[s^{\text{T}}\left(\sigma \right)K_{m}\left(\sigma \right)e\left(\sigma \right)+s^{\text{T}}\left(\sigma \right)D_{m}\left(\sigma \right)\dot{e}\left(\sigma \right)+s^{\text{T}}\left(\sigma \right)\tau_{m}\left(\sigma \right)+s^{\text{T}}\left(\sigma \right)\tau_{h}\left(\sigma \right)\right]d\sigma \leq 0 \end{array}.$$ (12)

In this mode, Eq. 3 can be written as Eq. 13.

$$\tau_{r}=M{\ddot{q}}_{\mathrm{ref}}+C{\dot{q}}_{\mathrm{ref}}+G+K\left(t\right)e+D\left(t\right)\dot{e}+L\left(t\right)s+\tau_{c}\left(t\right),$$ (13)

where the update rules of *K*(*t*), *D*(*t*), *L*(*t*), and $\tau_{c}\left(t\right)$ adhere to the following principles. 1) When $r_{j}^{\mathrm{par}}$ is negative and its absolute value is large, the patient’s motor ability is insufficient to complete the desired training task. In this case, the robot should increase its assistance level to help the patient complete training task. When $r_{j}^{\mathrm{par}}$ is negative and its absolute value is small, then, although the patient does not have the ability to complete the training task independently, the degree of active participation in the training is relatively high. In this case, the robot should reduce its assistance level to encourage the patient to further improve training enthusiasm. 2) The impedance parameters and torque compensation terms are adjusted adaptively by iterative learning. When the absolute value of $r_{j}^{\mathrm{ort}}$ is large, the learning speed increases to quickly correct the patient’s movement. When the absolute value of $r_{j}^{\mathrm{ort}}$ is small, the learning speed slows down.

The update law for *L*(*t*) is designed as follows:

$$\begin{array}{cc} L\left(t\right) & =\left(1+\eta_{j}\right)L_{0}\\ \eta_{j} & =\left(1+\eta \right)\left(1+\eta_{j-1}\right)-1, \end{array}$$ (14)

where

$$\left\{\begin{array}{cc} \eta =0,\; & r_{\min}^{\mathrm{par}}\leq r_{j}^{\mathrm{par}}\leq r_{\max}^{\mathrm{par}}\\ -1<\eta <0,\; & r_{j}^{\mathrm{par}}>r_{\max}^{\mathrm{par}}\\ 0<\eta <1,\; & r_{j}^{\mathrm{par}}<r_{\min}^{\mathrm{par}}, \end{array}\right.$$ (15)

where *η*_0_ = 0. *L*_0_ is a positive definite matrix. In this mode, $L\left(t\right)$ is periodically adjusted according to the value of $r_{j}^{\mathrm{par}}$ as shown in Eqs 14, 15. $r_{\min}^{\mathrm{par}}$ and $r_{\max}^{\mathrm{par}}$ are given constants, and their values are smaller than *c* and *d*. In practice, these two parameters can be adjusted according to the patient’s motor ability. If their motor ability is weak, $r_{\min}^{\mathrm{par}}$ and $r_{\max}^{\mathrm{par}}$ can be set to smaller values so that the controller can more easily detect the patient’s effort and reduce the robot’s assistance level.

The update law for *K*(*t*), *D*(*t*), and $\tau_{c}\left(t\right)$ are given as follows:

$$\left\{\begin{array}{cc} \Delta K\left(t\right) & =K\left(t\right)-K\left(t-T\right)=Q_{K}\left(se^{\text{T}}-\left(1+\gamma_{j}\right)K\left(t\right)\right)\\ \Delta D\left(t\right) & =D\left(t\right)-D\left(t-T\right)=Q_{D}\left(s{\dot{e}}^{\text{T}}-\left(1+\gamma_{j}\right)D\left(t\right)\right)\\ \Delta \tau_{c}\left(t\right) & =\tau_{c}\left(t\right)-\tau_{c}\left(t-T\right)=Q_{\tau_{c}}\left(s-\left(1+\gamma_{j}\right)\tau_{c}\left(t\right)\right) \end{array}\right.$$ (16)

where *γ*_*j*_ is updated as shown in Eq. 17.

$$\gamma_{j}=\left(1+\zeta \right)\left(1+\gamma_{j-1}\right)-1,\left\{\begin{array}{cc} \zeta =0,\; & r_{\min}^{\mathrm{ort}}\leq \left|r_{j}^{\mathrm{ort}}\right|\leq r_{\max}^{\mathrm{ort}}\\ -1<\zeta <0,\; & \left|r_{j}^{\mathrm{ort}}\right|>r_{\max}^{\mathrm{ort}}\\ 0<\zeta <1,\; & \left|r_{j}^{\mathrm{ort}}\right|<r_{\min}^{\mathrm{ort}} \end{array}\right.,$$ (17)

where $\gamma_{j}\in \left(-1,1\right)$ denotes the iterative learning factor, and *ζ* denotes the update rate. *γ*_0_ = 0. *Q*_*K*_, *Q*_*D*_, and $Q_{\tau_{c}}$ are symmetric positive definite matrices. During the first task cycle, *K*(*t*) = 0^*i*×*i*^, *D*(*t*) = 0^*i*×*i*^, and $\tau_{c}\left(t\right)=0^{i\times 1}$ .

In this mode, based on the assessment of motor ability and motor intention, $L\left(t\right)$ and *γ*_*j*_ are periodically adjusted to provide adaptive assistance for patients at different recovery stages.

### 3.3 The patient-dominant mode

When *α* = 0, *β* = 0, the controller is in the patient-dominant mode. In this mode, Eq. 3 can be written as Eq. 18.

$$\tau_{r}=M{\ddot{q}}_{\mathrm{ref}}+C{\dot{q}}_{\mathrm{ref}}+G+L\left(t\right)s+\tau_{c},$$ (18)

where $L\left(t\right)$ is given in Eq. 19.

$$L\left(t\right)=\left\{\begin{array}{cc} L_{0},\; & j=1\;or\;\lambda_{L_{\mathrm{last}}}<\lambda_{L_{0}}\\ L_{\mathrm{last}},\; & j>1\;and\;\lambda_{L_{\mathrm{last}}}\geq \lambda_{L_{0}} \end{array}\right.,$$ (19)

where *L*_*last*_ denotes the last updated value of *L*(*t*) before entering this mode. $\lambda_{L_{\mathrm{last}}}$ denotes the smallest eigenvalue of *L*_*last*_, and $\lambda_{L_{0}}$ denotes the smallest eigenvalue of *L*_0_. From the definition of ${\dot{q}}_{\mathrm{ref}}$ and *s*, we derive $s={\dot{q}}_{\mathrm{ref}}-\dot{q}={\dot{q}}_{d}+{\dot{q}}_{h}-\dot{q}$ . In this mode, ${\dot{q}}_{h}$ can be obtained by using the following impedance equation:

$$\tau_{h}=M_{im}{\ddot{q}}_{h}+B_{im}{\dot{q}}_{h}$$ (20)

where *M*_*im*_ and *B*_*im*_ denote the inertia and damping parameters, respectively.

**FIGURE 3:**
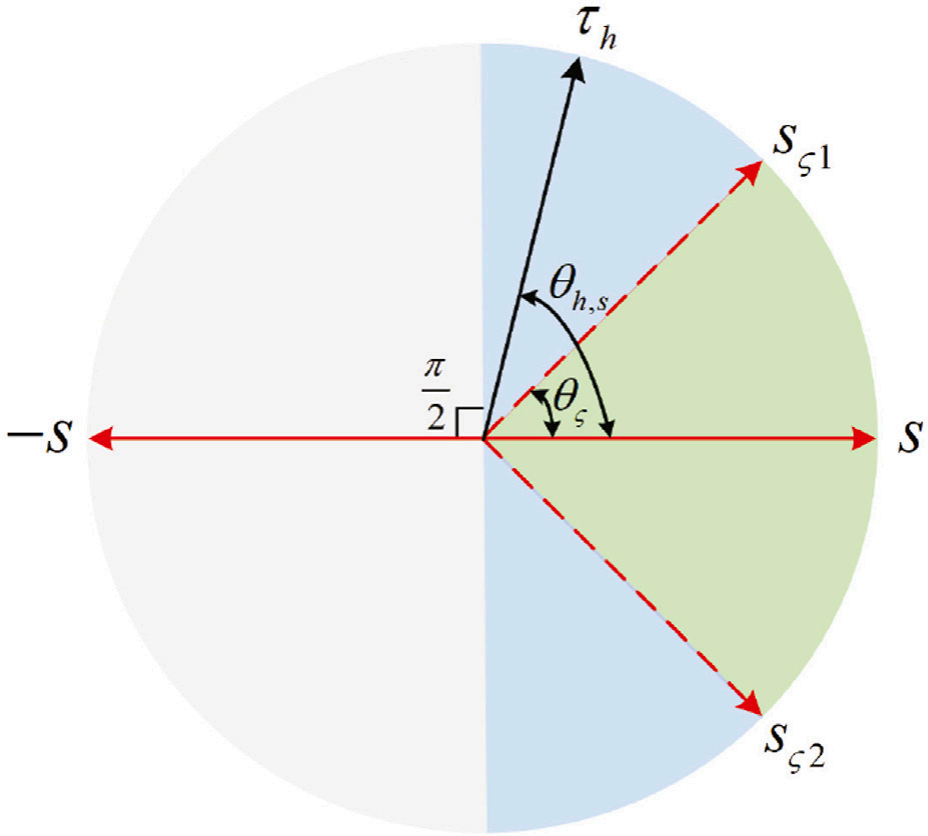
Schematic diagram of the compensation principle. When $\left|\theta_{h,s}\right|<\theta_{\varsigma }$ , *τ*_*h*_ is retained. When $\theta_{\varsigma }\leq \left|\theta_{h,s}\right|\leq \frac{\pi }{2}$ , *τ*_*h*_ is compensated to *s*_*ς*1_ or *s*_*ς*2_. When $\frac{\pi }{2}\leq \left|\theta_{h,s}\right|$ , *τ*_*c*_ is utilized to neutralize *τ*_*h*_.

To ensure the stability of human–robot interactions and encourage active patient participation, *τ*_*c*_ was utilized to appropriately compensate *τ*_*h*_ (Zhang and Cheah, 2015). When the absolute value of the angle *θ*_*h*,*s*_ between *τ*_*h*_ and *s* is smaller than *θ*_*ς*_ and $\theta_{\varsigma }\in \left(0,\frac{\pi }{2}\right)$ , then the patient exerts an interactive force to drive the robot close to the reference speed—that is, the patient’s motion intention can be seen as correct. In this case, *τ*_*h*_ is retained. When $\theta_{\varsigma }\leq \left|\theta_{h,s}\right|\leq \frac{\pi }{2}$ , then the patient’s motion intention cannot be seen as quite correct. In this case, *τ*_*h*_ is compensated to its nearest unit vector *s*_*ς*1_ or *s*_*ς*2_ to ensure that the angle between the compensated torque and *s* is equal to *θ*_*ς*_. When $\left|\theta_{h,s}\right|>\frac{\pi }{2}$ , the patient’s motion intention cannot be seen as correct. In this case, *τ*_*c*_ is utilized to neutralize *τ*_*h*_—that is, *τ*_*c*_ + *τ*_*h*_ = 0. The schematic diagram of the compensation principle is shown in Figure 3. In this mode, *τ*_*c*_ + *τ*_*h*_ can be expressed as Eqs 21−23

$$\tau_{c}+\tau_{h}=\mu \left(s\right)c\left(\tau_{h}\right),$$ (21)

where

$$\mu \left(s\right)=\left\{\begin{array}{cc} 1,\; & \left\|s\right\|\geq s_{\min}\\ \sin^{2}\left(\frac{\left\|s\right\|\pi }{2s_{\min}}\right),\; & \left\|s\right\|<s_{\min} \end{array}\right.$$ (22)

and

$$c\left(\tau_{h}\right)=\left\{\begin{array}{cc} \tau_{h},\; & \left|\theta_{h,s}\right|\in \left[0,\theta_{\varsigma }\right)\\ s_{\varsigma }\left\|\tau_{h}\right\|\cos^{2}\left(\frac{\left(\left|\theta_{h,s}\right|-\theta_{\varsigma }\right)\pi }{2\left(\frac{\pi }{2}-\theta_{\varsigma }\right)}\right),\; & \left|\theta_{h,s}\right|\in \left[\theta_{\varsigma },\frac{\pi }{2}\right]\\ 0,\; & \left|\theta_{h,s}\right|\in \left(\frac{\pi }{2},\pi \right], \end{array}\right.$$ (23)

where *s*_min_ is a small positive number. $\mu \left(s\right)$ ensures the smoothness of *τ*_*c*_ + *τ*_*h*_ at *s* = 0. *s*_*ς*_ equals *s*_*ς*1_ or *s*_*ς*2_.

In this mode, the impedance learning term is removed, and the sliding mode control term is converted to a speed control term. In addition, the patient can modify the reference speed, improving compliance with and the flexibility of rehabilitation training. *τ*_*c*_ is used to compensate *τ*_*h*_ appropriately. Compared with the robot-dominant mode, the patient-dominant mode further improves the patient’s freedom of movement.

### 3.4 The safety-stop mode

When *α* = 0, *β* = 1, the controller is in the safety-stop mode. In this mode, Eq. 3 can be written as Eq. 24.

$$\tau_{r}=M{\ddot{q}}_{\mathrm{ref}}+C{\dot{q}}_{\mathrm{ref}}+G+L\left(t\right)s+\tau_{c},$$ (24)

where $L\left(t\right)$ is given as Eq. 25.

$$L\left(t\right)=\left\{\begin{array}{cc} L_{0},\; & j=1\;or\;\lambda_{L_{\mathrm{last}}}<\lambda_{L_{0}}\\ L_{\mathrm{last}},\; & j>1\;and\;\lambda_{L_{\mathrm{last}}}\geq \lambda_{L_{0}}. \end{array}\right.$$ (25)

*τ*_*c*_ is utilized to neutralize *τ*_*h*_—that is, *τ*_*c*_ + *τ*_*h*_ = 0. From the definition of ${\dot{q}}_{\mathrm{ref}}$ and *s*, we derive $s={\dot{q}}_{\mathrm{ref}}-\dot{q}=-\dot{q}$ .

In this mode, the impedance learning term is removed, and the sliding mode control term is converted to a damping control term. The robot stops moving to ensure the patient’s safety.

## 4 Stability analysis

In this section, the Lyapunov stability theorem is used to establish the stability of the human–robot interaction process. Specifically, in the robot-dominant mode, *s* is limited to a certain bound. Under the assumption of Eq. (12), the learning errors of impedance parameters and torque compensation terms are bounded (Han et al., 2023). In the patient-dominant mode, the robot’s speed converges to ${\dot{q}}_{d}+{\dot{q}}_{h}$ . When it switches to the safety-stop mode, the robot’s speed decreases to zero.

The Lyapunov candidate function is chosen as Eq. 26.

$$V\left(t\right)=V_{1}\left(t\right)+V_{2}\left(t\right)=\frac{1}{2}s^{\text{T}}Ms+\frac{1}{2}\int_{t-T}^{t}\alpha {\tilde{\Psi }}^{\text{T}}\left(\sigma \right)Q^{-1}\tilde{\Psi }\left(\sigma \right)d\sigma ,$$ (26)

where

$$\tilde{\Psi }\left(t\right)=\Psi \left(t\right)-\Psi^{*}\left(t\right)={\left[vec{\left(\tilde{K}\left(t\right)\right)}^{\text{T}},vec{\left(\tilde{D}\left(t\right)\right)}^{\text{T}},{{\tilde{\tau }}_{c}\left(t\right)}^{\text{T}}\right]}^{\text{T}},$$ (27)

$$\left\{\begin{array}{c} \tilde{K}\left(t\right)=K\left(t\right)-K_{m}\left(t\right)\;\\ \tilde{D}\left(t\right)=D\left(t\right)-D_{m}\left(t\right)\;\\ {\tilde{\tau }}_{c}\left(t\right)=\tau_{c}\left(t\right)-\tau_{m}\left(t\right),\; \end{array}\right.$$ (28)

$$\left\{\begin{array}{c} \Psi \left(t\right)={\left[vec{\left(K\left(t\right)\right)}^{\text{T}},vec{\left(D\left(t\right)\right)}^{\text{T}},\tau_{c}{\left(t\right)}^{\text{T}}\right]}^{\text{T}}\;\\ \Psi^{*}\left(t\right)={\left[vec{\left(K_{m}\left(t\right)\right)}^{\text{T}},vec{\left(D_{m}\left(t\right)\right)}^{\text{T}},\tau_{m}{\left(t\right)}^{\text{T}}\right]}^{\text{T}}\;\\ Q=diag\left(I⊗Q_{K},I⊗Q_{D},Q_{\tau_{c}}\right),\; \end{array}\right.$$ (29)

where $vec\left(\cdot \right)$ represents the column vectorization operator. ⊗ represents the Kronecker product.

### 4.1 Stability analysis in the robot-dominant mode

In this mode, the system is stable if $V\left(t\right)$ is non-growing in each task cycle (Han et al., 2023).

$$\Delta V=V\left(t\right)-V\left(t-T\right)\leq 0.$$ (30)

Taking the derivative of $V_{1}\left(t\right)$ , we derive

$${\dot{V}}_{1}\left(t\right)=s^{\text{T}}M\dot{s}+\frac{1}{2}s^{\text{T}}\dot{M}s.$$ (31)

Since $\dot{M}-2C$ is an antisymmetric matrix, we can obtain Eq. 32.

$${\dot{M}}^{\text{T}}+\dot{M}=2\dot{M}=2C^{\text{T}}+2C.$$ (32)

Combining Eq. 31 and the definition of *s*, we then have

$$\begin{array}{c} {\dot{V}}_{1}\left(t\right)=s^{\text{T}}\left(M{\ddot{q}}_{\mathrm{ref}}-M\ddot{q}\right)+\frac{1}{2}s^{\text{T}}\left(C^{\text{T}}+C\right)s\\ =-\alpha s^{\text{T}}K\left(t\right)e-\alpha s^{\text{T}}D\left(t\right)\dot{e}-s^{\text{T}}L\left(t\right)s-s^{\text{T}}\tau_{c}\left(t\right)-s^{\text{T}}\tau_{h}\left(t\right). \end{array}$$ (33)

Since *α* = 1, *β* = 0, Eq. 33 can be expressed as Eq. 34.

$${\dot{V}}_{1}\left(t\right)=-s^{\text{T}}K\left(t\right)e-s^{\text{T}}D\left(t\right)\dot{e}-s^{\text{T}}L\left(t\right)s-s^{\text{T}}\tau_{c}\left(t\right)-s^{\text{T}}\tau_{h}\left(t\right).$$ (34)

Then, we can get Eq. 35

$$\begin{array}{cc} \Delta V_{1} & =\Delta V_{1}\left(t\right)-\Delta V_{1}\left(t-T\right)\\ & =\int_{t-T}^{t}-s^{\text{T}}K\left(\sigma \right)e-s^{\text{T}}D\left(\sigma \right)\dot{e}-s^{\text{T}}L\left(\sigma \right)s-s^{\text{T}}\tau_{c}\left(\sigma \right)-s^{\text{T}}\tau_{h}\left(\sigma \right)d\sigma \end{array}.$$ (35)

Since *L*(*t*) is periodically adjusted, the following inequality can be obtained:

$$\Delta V_{1}\leq \int_{t-T}^{t}-s^{\text{T}}K\left(\sigma \right)e-s^{\text{T}}D\left(\sigma \right)\dot{e}-s^{\text{T}}\lambda_{L}s-s^{\text{T}}\tau_{c}\left(\sigma \right)-s^{\text{T}}\tau_{h}\left(\sigma \right)d\sigma ,$$ (36)

where *λ*_*L*_ is the smallest eigenvalue of *L*(*σ*).

According to Eqs 12, 28, 36, we thus obtain Eq. 37

$$\begin{array}{cc} & \int_{t-T}^{t}-s^{\text{T}}K\left(\sigma \right)e-s^{\text{T}}D\left(\sigma \right)\dot{e}-s^{\text{T}}\lambda_{L}s-s^{\text{T}}\tau_{c}\left(\sigma \right)-s^{\text{T}}\tau_{h}\left(\sigma \right)d\sigma \\ & =\int_{t-T}^{t}\left[-s^{\text{T}}\tilde{K}\left(\sigma \right)e-s^{\text{T}}\tilde{D}\left(\sigma \right)\dot{e}-s^{\text{T}}\lambda_{L}s-s^{\text{T}}{\tilde{\tau }}_{c}\left(\sigma \right)\right.\\ & \left.-s^{\text{T}}K_{m}\left(\sigma \right)e-s^{\text{T}}D_{m}\left(\sigma \right)\dot{e}-s^{\text{T}}\tau_{m}\left(\sigma \right)-s^{\text{T}}\tau_{h}\left(\sigma \right)\right]d\sigma \\ & \leq \int_{t-T}^{t}\left[-s^{\text{T}}\tilde{K}\left(\sigma \right)e-s^{\text{T}}\tilde{D}\left(\sigma \right)\dot{e}-s^{\text{T}}\lambda_{L}s-s^{\text{T}}{\tilde{\tau }}_{c}\left(\sigma \right)\right]d\sigma , \end{array}$$ (37)

that is,

$$\Delta V_{1}\leq \int_{t-T}^{t}\left[-s^{\text{T}}\tilde{K}\left(\sigma \right)e-s^{\text{T}}\tilde{D}\left(\sigma \right)\dot{e}-s^{\text{T}}\lambda_{L}s-s^{\text{T}}{\tilde{\tau }}_{c}\left(\sigma \right)\right]d\sigma .$$ (38)

According to Eqs 27–29, we obtain

$$\begin{array}{cc} \Delta V_{2} & =\Delta V_{2}\left(t\right)-\Delta V_{2}\left(t-T\right)\\ & =\frac{1}{2}\int_{t-T}^{t}\underset{\mathrm{Item}\;a}{\underbrace{tr\left\{{\tilde{K}}^{\text{T}}\left(\sigma \right)Q_{K}^{-1}\tilde{K}\left(\sigma \right)-{\tilde{K}}^{\text{T}}\left(\sigma -T\right)Q_{K}^{-1}\tilde{K}\left(\sigma -T\right)\right\}}}\\ & +\underset{\mathrm{Item}\;b}{\underbrace{tr\left\{{\tilde{D}}^{\text{T}}\left(\sigma \right)Q_{D}^{-1}\tilde{D}\left(\sigma \right)-{\tilde{D}}^{\text{T}}\left(\sigma -T\right)Q_{D}^{-1}\tilde{D}\left(\sigma -T\right)\right\}}}\\ & +\underset{\mathrm{Item}\;c}{\underbrace{{{\tilde{\tau }}_{c}}^{\text{T}}\left(\sigma \right)Q_{\tau_{c}}^{-1}{\tilde{\tau }}_{c}\left(\sigma \right)-{{\tilde{\tau }}_{c}}^{\text{T}}\left(\sigma -T\right)Q_{\tau_{c}}^{-1}{\tilde{\tau }}_{c}\left(\sigma -T\right)}}d\sigma . \end{array}$$ (39)

Since $K_{m}\left(t\right)$ , $D_{m}\left(t\right)$ , and $\tau_{m}\left(t\right)$ are periodic, Eq. 16 can be written as Eq. 40.

$$\left\{\begin{array}{cc} \Delta K\left(t\right) & =K\left(t\right)-K\left(t-T\right)-K_{m}\left(t\right)+K_{m}\left(t-T\right)\\ & =\Delta \tilde{K}\left(t\right)=Q_{K}\left(se^{\text{T}}-\left(1+\gamma_{j}\right)K\left(t\right)\right)\\ \Delta D\left(t\right) & =D\left(t\right)-D\left(t-T\right)-D_{m}\left(t\right)+D_{m}\left(t-T\right)\\ & =\Delta \tilde{D}\left(t\right)=Q_{D}\left(s{\dot{e}}^{\text{T}}-\left(1+\gamma_{j}\right)D\left(t\right)\right)\\ \Delta \tau_{c}\left(t\right) & =\tau_{c}\left(t\right)-\tau_{c}\left(t-T\right)-\tau_{m}\left(t\right)+\tau_{m}\left(t-T\right)\\ & =\Delta {\tilde{\tau }}_{c}\left(t\right)=Q_{\tau_{c}}\left(s-\left(1+\gamma_{j}\right)\tau_{c}\left(t\right)\right) \end{array}\right.$$ (40)

Since $Q_{K}^{-1}$ is symmetric, *Item a* in Eq. 39 can be expressed thus:

$$\begin{array}{cc} & tr\left\{{\tilde{K}}^{\text{T}}\left(\sigma \right)Q_{K}^{-1}\tilde{K}\left(\sigma \right)-{\tilde{K}}^{\text{T}}\left(\sigma -T\right)Q_{K}^{-1}\tilde{K}\left(\sigma -T\right)\right\}\\ & =tr\left\{{\left[\tilde{K}\left(\sigma \right)-\tilde{K}\left(\sigma -T\right)\right]}^{\text{T}}Q_{K}^{-1}\left[\tilde{K}\left(\sigma \right)+\tilde{K}\left(\sigma -T\right)\right]\right\}\\ & =tr\left\{\Delta \tilde{K}{\left(\sigma \right)}^{\text{T}}Q_{K}^{-1}\left[2\tilde{K}\left(\sigma \right)-\left(\tilde{K}\left(\sigma \right)-\tilde{K}\left(\sigma -T\right)\right)\right]\right\}\\ & =tr\left\{-\Delta \tilde{K}{\left(\sigma \right)}^{\text{T}}Q_{K}^{-1}\Delta \tilde{K}\left(\sigma \right)+2\Delta \tilde{K}{\left(\sigma \right)}^{\text{T}}Q_{K}^{-1}\tilde{K}\left(\sigma \right)\right\}\\ & =-tr\left\{\Delta \tilde{K}{\left(\sigma \right)}^{\text{T}}Q_{K}^{-1}\Delta \tilde{K}\left(\sigma \right)\right\}+2tr\left\{{\left(se^{\text{T}}-\left(1+\gamma_{j}\right)K\left(\sigma \right)\right)}^{\text{T}}{Q^{\text{T}}}_{K}Q_{K}^{-1}\tilde{K}\left(\sigma \right)\right\}\\ & =-tr\left\{\Delta {\tilde{K}}^{\text{T}}\left(\sigma \right)Q_{K}^{-1}\Delta \tilde{K}\left(\sigma \right)\right\}+2tr\left\{{\left(se^{\text{T}}-\left(1+\gamma_{j}\right)K\left(\sigma \right)\right)}^{\text{T}}\tilde{K}\left(\sigma \right)\right\}\\ & =-tr\left\{\Delta {\tilde{K}}^{\text{T}}\left(\sigma \right)Q_{K}^{-1}\Delta \tilde{K}\left(\sigma \right)\right\}+2s^{\text{T}}\tilde{K}\left(\sigma \right)e-2\left(1+\gamma_{j}\right)tr\left\{K^{\text{T}}\left(\sigma \right)\tilde{K}\left(\sigma \right)\right\}. \end{array}$$ (41)

Similarly, *Item b* and *Item c* in Eq. 39 can be expressed as follows:

$$\begin{array}{c} tr\left\{{\tilde{D}}^{\text{T}}\left(\sigma \right)Q_{D}^{-1}\tilde{D}\left(\sigma \right)-{\tilde{D}}^{\text{T}}\left(\sigma -T\right)Q_{D}^{-1}\tilde{D}\left(\sigma -T\right)\right\}\\ =-tr\left\{\Delta {\tilde{D}}^{\text{T}}\left(\sigma \right)Q_{D}^{-1}\Delta \tilde{D}\left(\sigma \right)\right\}+2s^{\text{T}}\tilde{D}\left(\sigma \right)\dot{e}-2\left(1+\gamma_{j}\right)tr\left\{D^{\text{T}}\left(\sigma \right)\tilde{D}\left(\sigma \right)\right\} \end{array}$$ (42)

and

$$\begin{array}{c} {{\tilde{\tau }}_{c}}^{\text{T}}\left(\sigma \right)Q_{\tau_{c}}^{-1}{\tilde{\tau }}_{c}\left(\sigma \right)-{{\tilde{\tau }}_{c}}^{\text{T}}\left(\sigma -T\right)Q_{\tau_{c}}^{-1}{\tilde{\tau }}_{c}\left(\sigma -T\right)\\ =-\Delta {{\tilde{\tau }}_{c}}^{\text{T}}\left(\sigma \right)Q_{\tau_{c}}^{-1}\Delta {\tilde{\tau }}_{c}\left(\sigma \right)+2s^{\text{T}}{\tilde{\tau }}_{c}\left(\sigma \right)-2\left(1+\gamma_{j}\right){\tau_{c}}^{\text{T}}\left(\sigma \right){\tilde{\tau }}_{c}\left(\sigma \right). \end{array}$$ (43)

By bringing Eqs 41–43 into Eq. 39, we obtain

$$\begin{array}{cc} \Delta V_{2} & =\Delta V_{2}\left(t\right)-\Delta V_{2}\left(t-T\right)=-\frac{1}{2}\int_{t-T}^{t}\Delta {\tilde{\Psi }}^{\text{T}}\left(\sigma \right)Q^{-1}\Delta \tilde{\Psi }\left(\sigma \right)d\sigma \\ & +\int_{t-T}^{t}s^{\text{T}}\tilde{K}\left(\sigma \right)e+s^{\text{T}}\tilde{D}\left(\sigma \right)\dot{e}+s^{\text{T}}{\tilde{\tau }}_{c}\left(\sigma \right)d\sigma -\left(1+\gamma_{j}\right)\int_{t-T}^{t}{\tilde{\Psi }}^{\text{T}}\left(\sigma \right)\Psi \left(\sigma \right)d\sigma . \end{array}$$ (44)

By bringing Eqs 38, 44 into Eq. 30, we obtain Eq. 45.

$$\begin{array}{cc} \Delta V & =\Delta V_{1}\left(t\right)+\Delta V_{2}\left(t\right)\\ & \leq \int_{t-T}^{t}-\frac{1}{2}\Delta {\tilde{\Psi }}^{\text{T}}\left(\sigma \right)Q^{-1}\Delta \tilde{\Psi }\left(\sigma \right)-s^{\text{T}}\lambda_{L}s-\left(1+\gamma_{j}\right){\tilde{\Psi }}^{\text{T}}\left(\sigma \right)\Psi \left(\sigma \right)d\sigma \end{array}.$$ (45)

Since $\tilde{\Psi }\left(\sigma \right)=\Psi \left(\sigma \right)-\Psi^{*}\left(\sigma \right)$ , we can obtain Eq. 46

$$\begin{array}{cc} \Delta V & \leq \int_{t-T}^{t}-\frac{1}{2}\Delta {\tilde{\Psi }}^{\text{T}}\left(\sigma \right)Q^{-1}\Delta \tilde{\Psi }\left(\sigma \right)-s^{\text{T}}\lambda_{L}s\\ & -\left(1+\gamma_{j}\right){\tilde{\Psi }}^{\text{T}}\left(\sigma \right)\tilde{\Psi }\left(\sigma \right)-\left(1+\gamma_{j}\right){\tilde{\Psi }}^{\text{T}}\left(\sigma \right)\Psi^{*}\left(\sigma \right)d\sigma . \end{array}$$ (46)

A sufficient condition for Δ*V* to be non-positive definite is

$$\begin{array}{cc} & s^{\text{T}}\lambda_{L}s+\left(1+\gamma_{j}\right){\tilde{\Psi }}^{\text{T}}\left(\sigma \right)\tilde{\Psi }\left(\sigma \right)+\left(1+\gamma_{j}\right){\tilde{\Psi }}^{\text{T}}\left(\sigma \right)\Psi^{*}\left(\sigma \right)\\ & \geq \underset{\mathrm{Item}\;d}{\underbrace{\lambda_{L}{\left\|s\right\|}^{2}+\left(1+\gamma_{j}\right){\left\|\tilde{\Psi }\right\|}^{2}-\left(1+\gamma_{j}\right)\|\tilde{\Psi }\|\left\|\Psi^{*}\right\|}}\geq 0. \end{array}$$ (47)

When *Item d* in Eq. 47 is equal to zero, we obtain

$$\frac{{\left\|s\right\|}^{2}}{\left(1+\gamma_{j}\right){\left\|\Psi^{*}\right\|}^{2}/4\lambda_{L}}+\frac{{\left(\|\tilde{\Psi }\|-\left\|\Psi^{*}\right\|/2\right)}^{2}}{{\left\|\Psi^{*}\right\|}^{2}/4}=1.$$ (48)

According to LaSalle’s theorem, $\left\|s\right\|$ and $\left\|\tilde{\Psi }\right\|$ will converge on the invariant set Ω_*i*_ of Δ*V* = 0. Based on Eq. 48, a boundary set Ω can be designed as Eq. 49:

$$\Omega =\left\{\left(\left\|s\right\|,\|\tilde{\Psi }\|\right),\frac{{\left\|s\right\|}^{2}}{\left(1+\gamma_{j}\right){\left\|\Psi^{*}\right\|}^{2}/4\lambda_{L}}+\frac{{\left(\|\tilde{\Psi }\|-\left\|\Psi^{*}\right\|/2\right)}^{2}}{{\left\|\Psi^{*}\right\|}^{2}/4}\leq 1\right\}.$$ (49)

**FIGURE 4:**
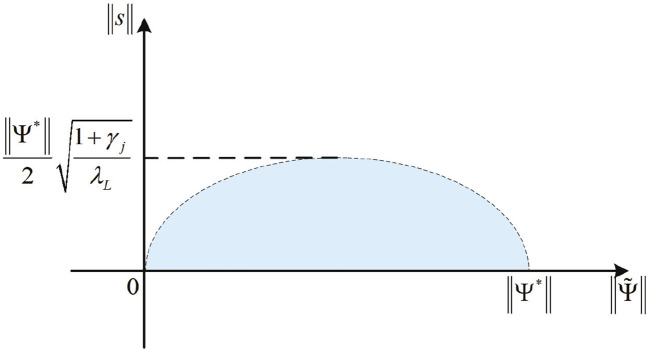
Schematic diagram of the boundary set Ω.

Since $\left\|s\right\|$ , $\left\|\tilde{\Psi }\right\|$ , and $\left\|\Psi^{*}\right\|$ are non-negative and 1 + *γ*_*j*_ and *λ*_*L*_ are positive numbers, the boundary set Ω is in the first quadrant, as shown in Figure 4.

From the inequality (Eq. 47), $\left\|s\right\|$ and $\left\|\tilde{\Psi }\right\|$ will converge on the invariant set Ω_*i*_ of Δ*V* = 0, and Ω_*i*_ ⊆Ω. *γ*_*j*_ and *L*(*t*) can be used to regulate the boundary set Ω. If *λ*_*L*_ increases, then a smaller $\left\|s\right\|$ is allowed, which means an increase in motion accuracy. If *λ*_*L*_ decreases, the system will allow for larger motion errors.

### 4.2 Stability analysis in the patient-dominant mode

In this mode, *α* = 0, *β* = 0. $V\left(t\right)$ and its derivative are expressed as follows.

$$V\left(t\right)=V_{1}\left(t\right)=\frac{1}{2}s^{\text{T}}Ms$$ (50)

and

$$\dot{V}\left(t\right)={\dot{V}}_{1}\left(t\right)=-s^{\text{T}}L\left(t\right)s-s^{\text{T}}\left(\tau_{c}+\tau_{h}\right)$$ (51)

By the definition of $L\left(t\right)$ , it is positive definite. From the definition of *τ*_*c*_ + *τ*_*h*_, the angle between *τ*_*c*_ + *τ*_*h*_ and *s* is less than or equal to $\frac{\pi }{2}$ —that is $s^{\text{T}}\left(\tau_{c}+\tau_{h}\right)\geq 0$ . Hence, we can get $\dot{V}\left(t\right)\leq 0$ , and $V\left(t\right)\leq V\left(0\right)$ . Since $V\left(0\right)$ is bounded, *s* is bounded.

To determine the consistent continuity of $\dot{V}\left(t\right)$ , Eq. 51 is derived as Eq. 52:

$$\ddot{V}\left(t\right)={\ddot{V}}_{1}\left(t\right)=-s^{\text{T}}L\left(t\right)\dot{s}-{\dot{s}}^{\text{T}}L\left(t\right)s-s^{\text{T}}\dot{L}\left(t\right)s-{\dot{s}}^{\text{T}}\left(\tau_{c}+\tau_{h}\right)-s^{\text{T}}\left({\dot{\tau }}_{c}+{\dot{\tau }}_{h}\right),$$ (52)

where $\dot{L}\left(t\right)=0$ . Due to the human motion ability limitation, *τ*_*h*_ and ${\dot{\tau }}_{h}$ can be assumed to be bounded. The boundedness of $\mu \left(s\right)$ and $c\left(\tau_{h}\right)$ ensures that *τ*_*c*_ + *τ*_*h*_ is bounded. ${\dot{\tau }}_{c}+{\dot{\tau }}_{h}$ can be expressed as Eq. 53

$${\dot{\tau }}_{c}+{\dot{\tau }}_{h}=\dot{\mu }\left(s\right)\dot{s}c\left(\tau_{h}\right)+\mu \left(s\right)\dot{c}\left(\tau_{h}\right){\dot{\tau }}_{h}.$$ (53)

The boundedness of *s* suggests the boundedness of ${\dot{q}}_{\mathrm{ref}}$ and $\dot{q}$ . Since ${\dot{q}}_{\mathrm{ref}}$ is bounded, ${\dot{q}}_{h}$ is too. According to Eq. 20, the boundedness of *τ*_*h*_ ensures that ${\ddot{q}}_{h}$ is bounded, so that ${\ddot{q}}_{\mathrm{ref}}$ is also bounded. The ${\dot{\tau }}_{c}+{\dot{\tau }}_{h}$ is bounded due to the boundedness of $\dot{\mu }\left(s\right)$ , $\dot{s}$ , $c\left(\tau_{h}\right)$ , $\mu \left(s\right)$ , ${\dot{\tau }}_{h}$ , and $\dot{c}\left(\tau_{h}\right)$ . Therefore, $\ddot{V}\left(t\right)$ is bounded. According to Barbalat’s lemma, $\underset{t\to \infty }{\lim}\dot{V}\left(t\right)\to 0$ , which means that if *t* → *∞*, *s* → 0. From the definition of *s*, the robot’s speed converges to ${\dot{q}}_{\mathrm{ref}}$ —that is, ${\dot{q}}_{d}+{\dot{q}}_{h}$ .

### 4.3 Stability analysis in the safety-stop mode

When the trajectory error is too large, it will switch to the patient-dominant mode—*α* = 0, *β* = 1. $V\left(t\right)$ and its derivative are the same as Eqs 50, 51. In this mode, $L\left(t\right)$ is positive definite and *τ*_*c*_ + *τ*_*h*_ = 0; thus, we obtain $\dot{V}\left(t\right)\leq 0$ , and $V\left(t\right)\leq V\left(0\right)$ . Since $V\left(0\right)$ is bounded, *s* is bounded. The derivation of $\dot{V}$ is given as Eq. 54

$$\ddot{V}\left(t\right)={\ddot{V}}_{1}\left(t\right)=-s^{\text{T}}L\left(t\right)\dot{s}-{\dot{s}}^{\text{T}}L\left(t\right)s-s^{\text{T}}\dot{L}\left(t\right)s,$$ (54)

where $\dot{L}\left(t\right)=0$ . The boundedness of *τ*_*r*_ + *τ*_*h*_ ensures the boundedness of $\ddot{q}$ and $\dot{s}$ . Therefore, $\ddot{V}\left(t\right)$ is bounded. According to Barbalat’s lemma, $\underset{t\to \infty }{\lim}\dot{V}\left(t\right)\to 0$ , so that if *t* → *∞*, *s* → 0. From the definition of *s*, the robot will stop moving.

## 5 Simulations

**FIGURE 5:**
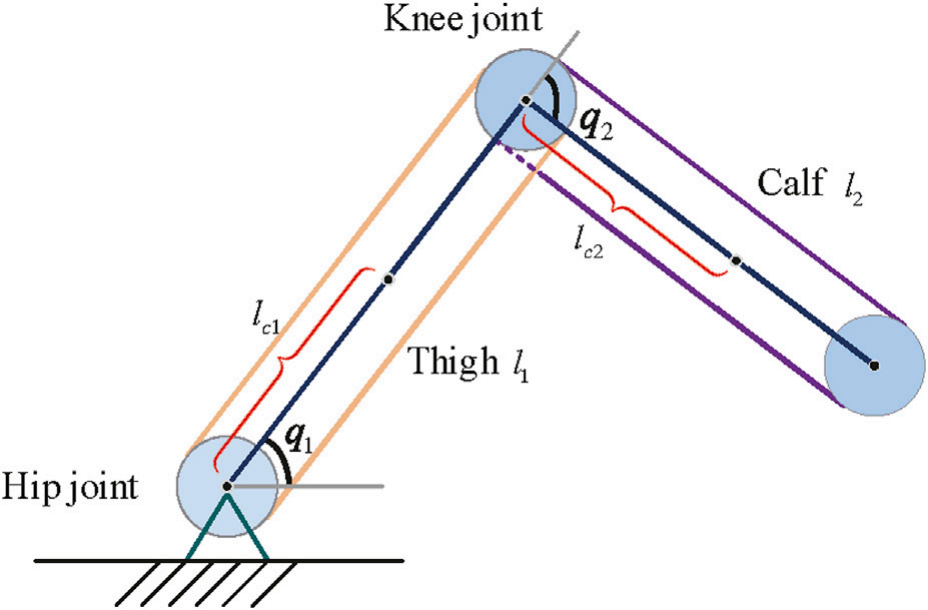
Simplified structure of a lower limb rehabilitation robot.

A two-degree-of-freedom lower limb rehabilitation robot is used to verify the effectiveness of the proposed method. As shown in Figure 5, *m*_1_ and *m*_2_ represent the mass of the thigh and calf, respectively. *l*_1_ and *l*_2_ represent the length of the thigh and calf, respectively. *l*_*c*1_ denotes the distance from the hip joint to the center of mass of the thigh. *l*_*c*2_ denotes the distance from knee joint to the center of mass of the calf. The dynamic model of this hybrid system is described as Eq. 55

$$\left[\begin{array}{cc} M_{11} & M_{12}\\ M_{21} & M_{22} \end{array}\right]\left[\begin{array}{c} {\ddot{q}}_{1}\\ {\ddot{q}}_{2} \end{array}\right]+\left[\begin{array}{cc} C_{11} & C_{12}\\ C_{21} & C_{22} \end{array}\right]\left[\begin{array}{c} {\dot{q}}_{1}\\ {\dot{q}}_{2} \end{array}\right]+\left[\begin{array}{c} G_{1}\\ G_{2} \end{array}\right]=\left[\begin{array}{c} \tau_{r,1}\\ \tau_{r,2} \end{array}\right]+\left[\begin{array}{c} \tau_{h,1}\\ \tau_{h,2} \end{array}\right],$$ (55)

where $M_{11}=I_{1}+I_{2}+m_{2}l_{1}^{2}+2m_{2}l_{1}l_{c2}cos\left(q_{2}\right)$ . $I_{1}=m_{1}l_{c1}^{2}$ and $I_{2}=m_{2}l_{c2}^{2}$ represent the inertia of the thigh and calf, respectively. $M_{12}=I_{2}+m_{2}l_{1}l_{c2}cos\left(q_{2}\right)$ , *M*_21_ = *M*_12_, *M*_22_ = *I*_2_, $C_{11}=-C_{0}{\dot{q}}_{2}$ , $C_{12}=-C_{0}\left({\dot{q}}_{1}+{\dot{q}}_{2}\right)$ , $C_{21}=C_{0}{\dot{q}}_{1}$ , *C*_22_ = 0, and $C_{0}=m_{2}l_{1}l_{c2}sin\left(q_{2}\right)$ . *G*_1_ = (*m*_1_ *l*_*c*1_ + *m*_2_ *l*_1_)*gcos*(*q*_1_) + *m*_2_ *l*_*c*2_ *gcos*(*q*_1_ + *q*_2_). *G*_2_ = *m*_2_ *l*_*c*2_ *gcos*(*q*_1_ + *q*_2_). *g* is the acceleration of gravity. The desired trajectory is designed as Eq. 56.

$$\left\{\begin{array}{cc} q_{d,1} & =\frac{\pi }{6}-\frac{\pi }{12}cos\left(0.2\pi t\right)\\ q_{d,2} & =-\frac{\pi }{3}+\frac{\pi }{9}sin\left(0.2\pi t\right) \end{array}\right.$$ (56)

The initial angle of the robot is set to $q_{0}={\left[7\pi /36,-13\pi /36\right]}^{\text{T}}$ . The initial parameters of the proposed method and lower limb rehabilitation robot are listed in Table 1. The values of *η* and *ζ* are given as Eqs 57, 58

$$\left\{\begin{array}{cc} \eta =0,\; & -2\leq r_{j}^{\mathrm{par}}\leq -1\\ \eta =-0.2,\; & r_{j}^{\mathrm{par}}>-1\\ \eta =0.2,\; & r_{j}^{\mathrm{par}}<-2, \end{array}\right.$$ (57)

$$\left\{\begin{array}{cc} \zeta =0,\; & 0.1\leq \left|r_{j}^{\mathrm{ort}}\right|\leq 0.5\\ \zeta =-0.3,\; & \left|r_{j}^{\mathrm{ort}}\right|>0.5\\ \zeta =0.3,\; & \left|r_{j}^{\mathrm{ort}}\right|<0.1. \end{array}\right.$$ (58)

**TABLE 1:** Initialization parameters in simulation.

Parameter	Value	Parameter	Value
*a*	*π*/12	$Q_{\tau_{c}}$	$\left[30,0;0,30\right]$
*b*	*π*/18	*s*_min_	0.008
*c*	−0.1	*θ*_*ς*_	4*π*/9
*d*	−0.4	$r_{1}^{\mathrm{par}}$	−1
*L*_0_	$\left[4,0;0,4\right]$	$r_{1}^{\mathrm{ort}}$	0
$r_{\min}^{\mathrm{par}}$	−2	*A*	$\left[10,0;0,10\right]$
$r_{\max}^{\mathrm{par}}$	−1	*m*_1_	8
$r_{\min}^{\mathrm{ort}}$	0.1	*m*_2_	8
$r_{\max}^{\mathrm{ort}}$	0.5	*l*_1_	0.5
*Q*_*K*_	$\left[50,0;0,50\right]$	*l*_2_	0.6
*Q*_*D*_	$\left[10,0;0,10\right]$	*l*_*c*1_	0.3
*T*	10	*l*_*c*2_	0.4
*M*_*im*_	$\left[120,0;0,60\right]$	*T*_*smo*_	1.7
*B*_*im*_	$\left[60,0;0,30\right]$		

**FIGURE 6:**
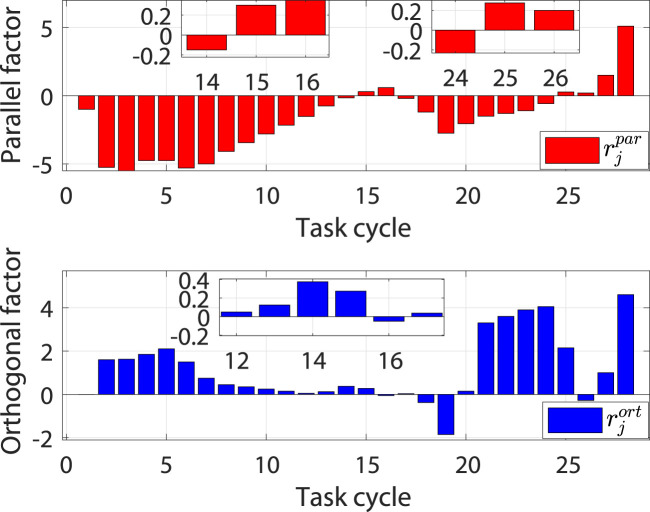
Evaluation results of the human–robot interaction force under different task cycles.

**FIGURE 7:**
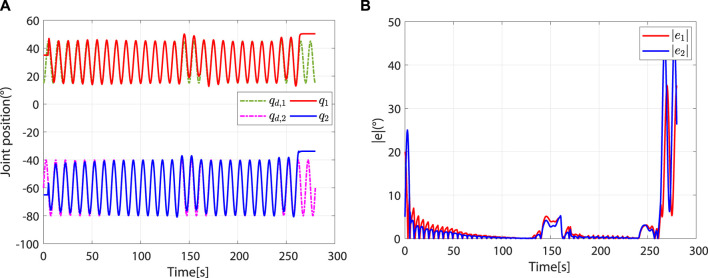
Entire simulation results of hip and knee joints. **(A)** Desired and actual trajectories. **(B)** Absolute values of trajectory errors.

The simulation process consists of 28 task cycles, each lasting 10 s. The results of the human–robot interaction force evaluation for each task cycle are given in Figure 6, which shows the patients’ motor ability and motion intention under different task cycles. The simulation results are shown in Figure 7.

**FIGURE 8:**
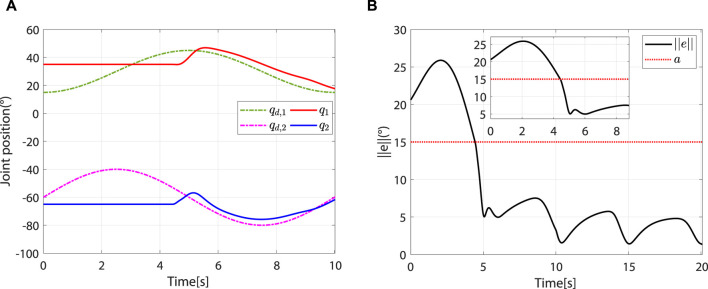
The controller leaves the safety-stop mode at approximately 4.44 s. **(A)** Desired and actual trajectories. **(B)** At approximately 4.44 s, $\left\|e\right\|<a$ .

At the beginning, the controller is in safety-stop mode due to $\left\|e\right\|>a$ . In this mode, *τ*_*h*_ is neutralized by *τ*_*c*_. At approximately 4.44 s, $\left\|e\right\|<a$ . Meanwhile, due to $r_{1}^{\mathrm{par}}=-1$ , the controller leaves the safety-stop mode and gradually transitions to the robot-dominant mode (Figure 8).

**FIGURE 9:**
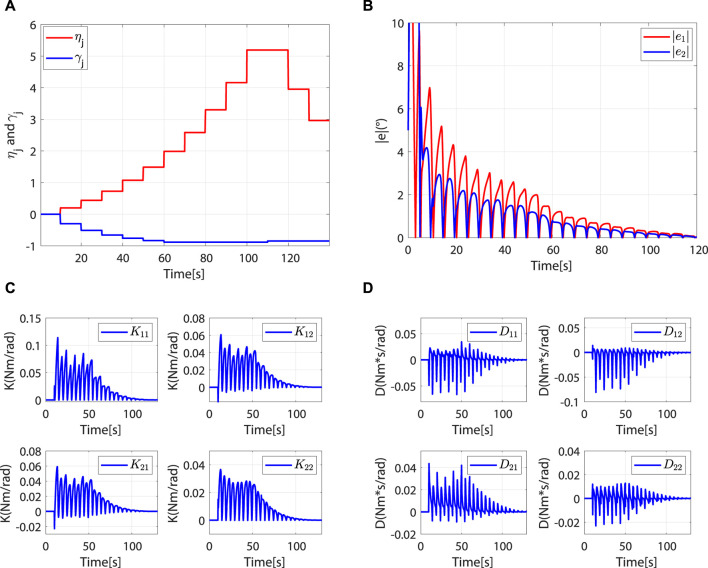
The controller is in the robot-dominant mode, and the robot assisted the patient to complete the rehabilitation task. **(A)** *η*_*j*_ and *γ*_*j*_. **(B)** Absolute values of trajectory errors. **(C)** *K*(*t*). **(D)** *D*(*t*).

In the robot-dominant mode, according to $r_{j}^{\mathrm{par}}$ and $r_{j}^{\mathrm{ort}}$ , the robot’s assistance level is adaptively adjusted to help the patient complete the desired task. The change trends of *η*_*j*_ and *γ*_*j*_ are shown in Figure 9A. When $r_{j}^{\mathrm{par}}<r_{\min}^{\mathrm{par}}$ , *L*(*t*) increases periodically with *η*_*j*_. Although there are fluctuations in the changes of $r_{j}^{\mathrm{par}}$ and $r_{j}^{\mathrm{ort}}$ , the trajectory error decreases periodically (Figure 9B). The increase of the eigenvalue of *L*(*t*) improves motion accuracy. When $r_{\min}^{\mathrm{par}}<r_{j}^{\mathrm{par}}<r_{\max}^{\mathrm{par}}$ , *η*_*j*_ remains unchanged. When $r_{j}^{\mathrm{par}}>r_{\max}^{\mathrm{par}}$ , *L*(*t*) decreases periodically with *η*_*j*_. It can be observed that $\left|r_{j}^{\mathrm{ort}}\right|$ is greater than $r_{\max}^{\mathrm{ort}}$ in the second to seventh task cycle, which means that the patient intends to move away from the desired trajectory. *γ*_*j*_ will then reduce to a lower value to increase the learning rate of the impedance parameters, thus correcting the patient’s motion trajectory (Figure 9A). However, with the gradual reduction of *γ*_*j*_, the impedance parameters present a tendency to decrease periodically (Figures 9C, D). According to Eq. 16, this phenomenon is attributed to the improvement of motion accuracy.

**FIGURE 10:**
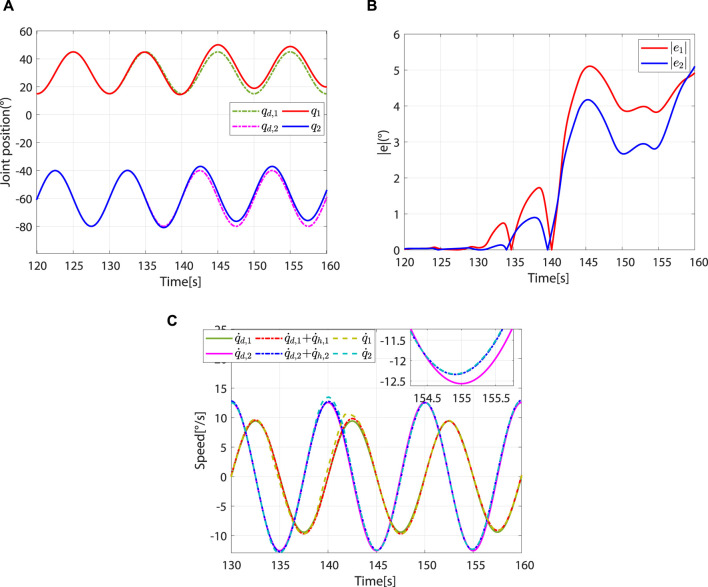
Controller switches from robot-dominant to patient-dominant mode and remains in the patient-dominant mode. **(A)** Desired and actual trajectories. **(B)** Absolute values of the trajectory errors. **(C)** Desired angular speed, reference angular speed, and actual angular speed.

When $d<r_{j}^{\mathrm{par}}\leq c$ , the controller breaks away from the robot-dominant mode and transitions to the patient-dominant mode. When $r_{j}^{\mathrm{par}}>c$ , the controller is in patient-dominant mode. As the controller switches from robot-dominant to patient-dominant mode, greater trajectory errors are allowed, which provides greater freedom of movement (Figures 10A, B). In addition, the robot’s speed gradually converges to ${\dot{q}}_{d}+{\dot{q}}_{h}$ (Figure 10C). This phenomenon is consistent with theory.

**FIGURE 11:**
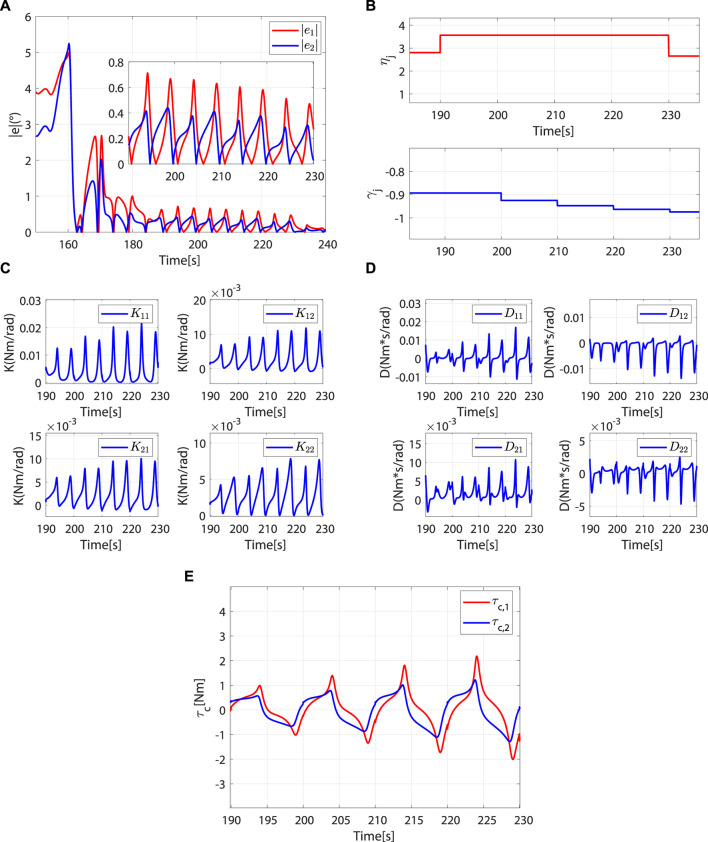
Controller switches from patient-dominant to robot-dominant mode and remains in the robot-dominant mode. **(A)** Absolute values of trajectory errors. **(B)** *η*_*j*_ and *γ*_*j*_. **(C)** *K*(*t*). **(D)** *D*(*t*). **(E)** *τ*_*c*_(*t*).

As $r_{j}^{\mathrm{par}}$ decreases, the controller switches again to the robot-dominant mode. Compared to the patient-dominant mode, the trajectory error is significantly reduced at this point (Figure 11A). From 190 to 230 s, *η*_*j*_ does not change. Affected by the vertical interaction force, *γ*_*j*_ decreases periodically (Figure 11B). From Figures 11C–E, the impedance parameter and torque compensation term increase periodically to correct patient motion, and the trajectory error is gradually reduced (Figure 11A).

**FIGURE 12:**
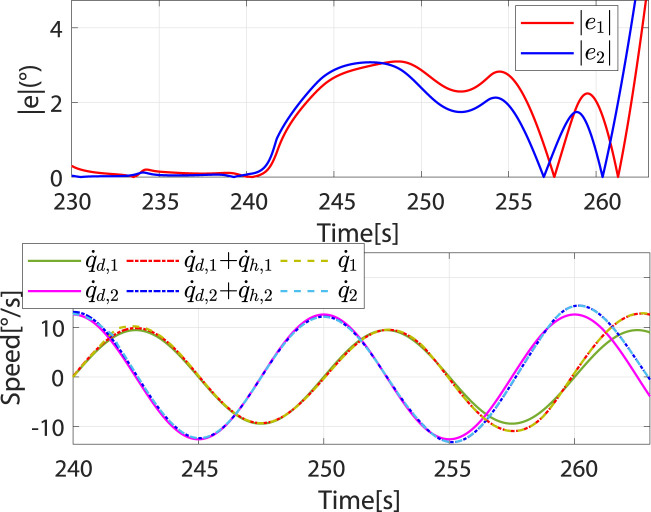
Speed and absolute values of trajectory errors when controller switches again from robot-dominant to patient-dominant mode.

**FIGURE 13:**
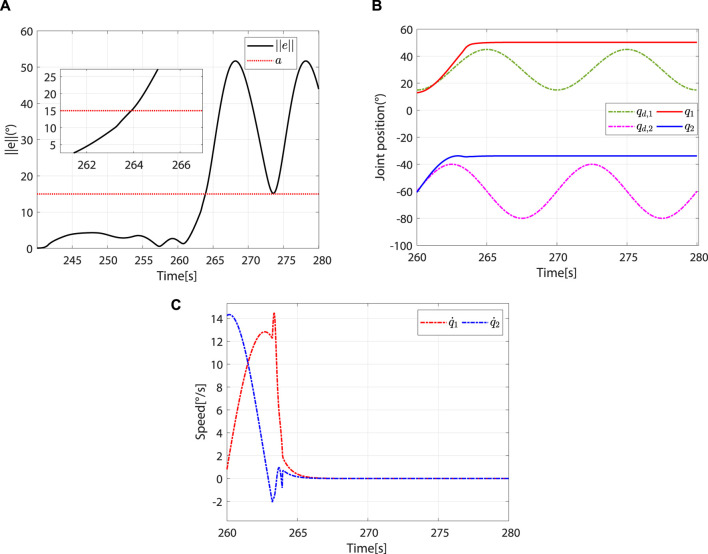
The controller switches to the safety-stop mode. **(A)** At approximately 263.92 s, $\left\|e\right\|>a$ . **(B)** Desired and actual trajectories. **(C)** Actual angular speed.

When $r_{j}^{\mathrm{par}}>c$ , the controller switches to the patient-dominant mode again, where the patient’s freedom of movement increases and the robot’s speed converges to ${\dot{q}}_{d}+{\dot{q}}_{h}$ (Figure 12). To test the safety stop function of the controller during rehabilitation training, the excessive $F_{h}^{\mathrm{par}}$ and $F_{h}^{\mathrm{ort}}$ are applied, which will cause $\left\|e\right\|$ to increase sharply and $\left\|e\right\|>a$ (Figure 13A). In this case, the controller switches to the safety-stop mode to ensure patient safety (Figure 13B), and the actual speed of the robot decreases rapidly to zero (Figure 13C).

The effectiveness of the proposed method is demonstrated by the trajectory errors, adaptive change of controller parameters, and joint angular speed during human–robot interaction in three modes. In addition, the simulation includes the transition process between each mode, and the system can still run stably during this transition process.

## 6 Conclusion

This study proposes a multi-mode adaptive control method, including robot-dominant, patient-dominant, and safety-stop modes. The patient’s motor ability and the system’s trajectory error are taken as the basis for mode switching. Based on the patients’ motor ability, the controller can switch between robot-dominant and patient-dominant modes. Trajectory errors are used to determine whether to switch to the safety-stop mode. The proposed control strategy is not only suitable for patients with different motor abilities and rehabilitation stages but also guarantees safety during rehabilitation training. Since the transition between robot-dominant and patient-dominant modes does not depend on the trajectory errors, the patient-dominant mode allows for greater trajectory errors than the robot-dominated mode, and the reference speed can be modified by the patient, improving their freedom of movement. The stability of the proposed method under three control modes is analyzed using Lyapunov theory. Numerical simulations are carried out on a two-degree-of-freedom lower limb rehabilitation robot to verify the effectiveness of the proposed method. Our future work will focus on clinical applications.

## Data Availability

The original contributions presented in the study are included in the article/supplementary material; further inquiries can be directed to the corresponding author.

## References

[B1] Adhikari B. Bharadwaj V. R. Miller B. A. Novak V. D. Jiang C. (2023). Learning skill training schedules from domain experts for a multi-patient multi-robot rehabilitation gym. IEEE Trans. Neural Syst. Rehabil. Eng. 31, 4256–4265. 10.1109/TNSRE.2023.3326777 37871090

[B2] Asl H. J. Yamashita M. Narikiyo T. Kawanishi M. (2020). Field-based assist-as-needed control schemes for rehabilitation robots. IEEE ASME Trans. Mechatron. 25, 2100–2111. 10.1109/TMECH.2020.2992090

[B3] Bergmann L. Voss D. Leonhardt S. Ngo C. (2023). Lower-limb exoskeleton with compliant actuators: human cooperative control. IEEE Trans. Med. Robot. 5, 717–729. 10.1109/TMRB.2023.3290982

[B4] Gao M. Chen J. Li M. Dai J. S. (2023). “Design and evaluation of a novel self-adaptive ankle rehabilitation exoskeleton with elastic modules,” in 2023 international conference on advanced robotics and mechatronics (ICARM), 900–905. 10.1109/ICARM58088.2023.10218858

[B5] Guo L. Lu Z. Yao L. (2021). Human-machine interaction sensing technology based on hand gesture recognition: a review. IEEE Trans. Hum. Mach. Syst. 51, 300–309. 10.1109/THMS.2021.3086003

[B6] Han S. Wang H. Yu H. (2023). Human-robot interaction evaluation-based AAN control for upper limb rehabilitation robots driven by series elastic actuators. IEEE Trans. Robot. 39, 3437–3451. 10.1109/TRO.2023.3286073

[B7] Jamwal P. K. Hussain S. Ghayesh M. H. Rogozina S. V. (2016). Impedance control of an intrinsically compliant parallel ankle rehabilitation robot. IEEE Trans. Ind. Electron. 63, 3638–3647. 10.1109/TIE.2016.2521600

[B8] Li N. Yang Y. Li G. Yang T. Wang Y. Chen W. (2024a). Multi-sensor fusion-based mirror adaptive assist-as-needed control strategy of a soft exoskeleton for upper limb rehabilitation. IEEE Trans. Autom. 21, 475–487. 10.1109/TASE.2022.3225727

[B9] Li X. Pan Y. Chen G. Yu H. (2017a). Adaptive human-robot interaction control for robots driven by series elastic actuators. IEEE Trans. Robot. 33, 169–182. 10.1109/TRO.2016.2626479

[B10] Li X. Pan Y. Chen G. Yu H. (2017b). Multi-modal control scheme for rehabilitation robotic exoskeletons. Int. J. Robot. Res. 36, 759–777. 10.1177/0278364917691111

[B11] Li X. Yang Q. Song R. (2021). Performance-based hybrid control of a cable-driven upper-limb rehabilitation robot. IEEE Trans. Biomed. Eng. 68, 1351–1359. 10.1109/TBME.2020.3027823 32997619

[B12] Li Z. Zhang T. Huang P. Li G. (2024b). Human-in-the-loop cooperative control of a walking exoskeleton for following time-variable human intention. IEEE Trans. Cybern. 54, 2142–2154. 10.1109/TCYB.2022.3211925 36279358

[B13] Liang X. Su T. Zhang Z. Zhang J. Liu S. Zhao Q. (2022). An adaptive time-varying impedance controller for manipulators. Front. Neurorobot. 16, 789842. 10.3389/fnbot.2022.789842 35370593 PMC8971993

[B14] Liang X. Yan Y. Su T. Guo Z. Liu S. Zhang H. (2023). “Kalman filter and moving average method based human-robot interaction torque estimation for a lower limb rehabilitation robot,” in 2023 international conference on advanced robotics and mechatronics (ICARM), 1083–1088. 10.1109/ICARM58088.2023.10218932

[B15] Lu Z. He B. Cai Y. Chen B. Yao L. Huang H. (2023). Human-machine interaction technology for simultaneous gesture recognition and force assessment: a review. IEEE Sens. J. 23, 26981–26996. 10.1109/JSEN.2023.3314104

[B16] Luo L. Peng L. Wang C. Hou Z.-G. (2019). A greedy assist-as-needed controller for upper limb rehabilitation. IEEE Trans. Neural Netw. Learn. Syst. 30, 3433–3443. 10.1109/TNNLS.2019.2892157 30736008

[B17] Mao Y. Jin X. Gera Dutta G. Scholz J. P. Agrawal S. K. (2015). Human movement training with a cable driven arm exoskeleton (CAREX). IEEE Trans. Neural Syst. Rehabil. Eng. 23, 84–92. 10.1109/TNSRE.2014.2329018 24919202

[B18] Masengo G. Zhang X. Dong R. Alhassan A. B. Hamza K. Mudaheranwa E. (2023). Lower limb exoskeleton robot and its cooperative control: a review, trends, and challenges for future research. Front. Neurorobot. 16, 913748. 10.3389/fnbot.2022.913748 36714152 PMC9875327

[B19] Xu J. Li Y. Xu L. Peng C. Chen S. Liu J. (2019). A multi-mode rehabilitation robot with magnetorheological actuators based on human motion intention estimation. IEEE Trans. Neural Syst. Rehabil. Eng. 27, 2216–2228. 10.1109/TNSRE.2019.2937000 31443038

[B20] Yang C. Ganesh G. Haddadin S. Parusel S. Albu-Schaeffer A. Burdet E. (2011). Human-like adaptation of force and impedance in stable and unstable interactions. IEEE Trans. Robot. 27, 918–930. 10.1109/TRO.2011.2158251

[B21] Yang R. Shen Z. Lyu Y. Zhuang Y. Li L. Song R. (2023). Voluntary assist-as-needed controller for an ankle power-assist rehabilitation robot. IEEE Trans. Biomed. Eng. 70, 1795–1803. 10.1109/TBME.2022.3228070 37015472

[B22] Zhang J. Cheah C. C. (2015). Passivity and stability of human-robot interaction control for upper-limb rehabilitation robots. IEEE Trans. Robot. 31, 233–245. 10.1109/TRO.2015.2392451

[B23] Zhou J. Li Z. Li X. Wang X. Song R. (2021). Human-robot cooperation control based on trajectory deformation algorithm for a lower limb rehabilitation robot. IEEE ASME Trans. Mechatron. 26, 3128–3138. 10.1109/TMECH.2021.3053562

